# Understanding the Causes of High Organic Matter with Low Bioavailability in Cold-Zone Lake Water: A Case in Hulun Lake

**DOI:** 10.3390/toxics14040347

**Published:** 2026-04-20

**Authors:** Yulong Tao

**Affiliations:** Hulunbuir Academy of Inland Lakes in Northern Cold & Arid Areas, Hulunbuir 021000, China; taoyulong_1982@163.com

**Keywords:** dissolved organic matter (DOM), molecular composition, bioavailability, cold-zone lake

## Abstract

In cold-region lakes, high organic matter concentrations with low bioavailability are common, yet the underlying causes and stabilisation mechanisms remain unclear. This study conducted a 60-day microbial treatment experiment in Hulun Lake using algae (DOMa), grass (DOMg), and manure (DOMm) as DOM sources. Fourier transform ion cyclotron resonance mass spectrometry and 16S rRNA analysis were employed to characterise DOM composition and bacterial communities. The bioavailability of DOMa, DOMg, and DOMm was 86.1%, 84.08%, and 70.9%, respectively. Differences in degradation cycles were mainly associated with the slowly biodegradable fraction; the half-lives of DOMa, DOMg, and DOMm were 49.51 days, 77.02 days, and 198.04 days, respectively. At the molecular level, proteins and lipids were rapidly utilised by microorganisms, leading to the generation of lignin, condensed aromatic hydrocarbons, and tannins, with many new molecules falling within the carboxylic acid-rich alicyclic molecule (CRAM) region. The overall community succession patterns of different DOM sources were highly similar, with initial DOM composition differences leading to variations in microbial communities during intermediate degradation stages (5~10 days). Moreover, microbiological processes facilitated the convergence of DOM source compositions and the accumulation of refractory organic matter. It is hypothesised that the regional climatic characteristics of the freeze–thaw cycle exacerbate organic matter accumulation by compressing the “effective degradation time”. These findings elucidate the causes of high organic matter and low bioavailability in cold-region lakes.

## 1. Introduction

As one of the largest reservoirs of reactive carbon on Earth, dissolved organic matter (DOM) is critical in the biogeochemical cycling of carbon in aquatic ecosystems [1,2]. DOM is a major nutrient and carbon source that is involved in a variety of material cycling processes in lake ecosystems. The chemical properties of DOM in lakes are mainly determined by its sources and biogeochemical transport processes. The external sources are mainly from inflowing waters and atmospheric deposition, which include anthropogenic products in most cases [3,4]. The internal sources are mainly derived from metabolic processes and residues of aquatic plants, algae, bacteria and sediment releases [5]. However, external DOM inputs to most lakes have been increasing in recent decades due to both climate change and human activities [6]. Meanwhile, the increase in primary productivity of lakes further contributes to the production of internal sources of DOM. As a result, the stability of lake organic carbon is affected by changes in DOM sources to some extent [7].

The bioavailability of DOM, which is composed of a variety of organic compounds, mainly depends on the properties of the compounds and the interaction with the physical, chemical and biological environment [8,9]. The chemical composition of DOM often depends on its sources, which tend to control the overall reactivity of organic matter to a large extent. Autochthonous DOM is susceptible to microbial metabolism due to its richness in proteins and unstable polysaccharides [10,11], while terrestrial DOM contains more highly aromatic humic substances and is difficult for microorganisms to degrade [7,12]. Microbial communities with different structures differ considerably in the degradation of DOM [13], which is related to selective microbial preferences for carbon sources [9] and keystone species that respond to DOM [14]. In addition, bacterial degradation of DOM in aquatic ecosystems is a succession of reaction processes [15], in which the labile fraction is consumed first, followed by the semilabile fraction, while refractory pools persist in aquatic ecosystems [16]. Thus, the bioavailability of DOM may depend on the time of exposure of DOM substrates to aquatic ecosystems. The bioavailability of DOM is influenced by many factors; however, little is known about the causes of DOM characterisation and the associated processes in natural lakes, especially regarding different sources.

The fate and transport of DOM in cold-zone lakes are usually characterised by high concentrations [17,18], high biostability, and a high proportion of humus [19,20,21,22]. The lakes usually have 4–6 months of ice-covered period [20,23] in the winter, during which DOM is accumulated. Although the lakes have a shorter summer with higher air temperatures, algal blooms occur more frequently, and internal sources of DOM are not negligible, especially in shallow eutrophic lakes [24,25,26]. However, traditional carbon assessment methods [chemical oxygen demand (COD)] significantly underestimate the actual level of organic pollution in regional lakes, thus posing a significant challenge to local regulators [20].

**Figure 1:**
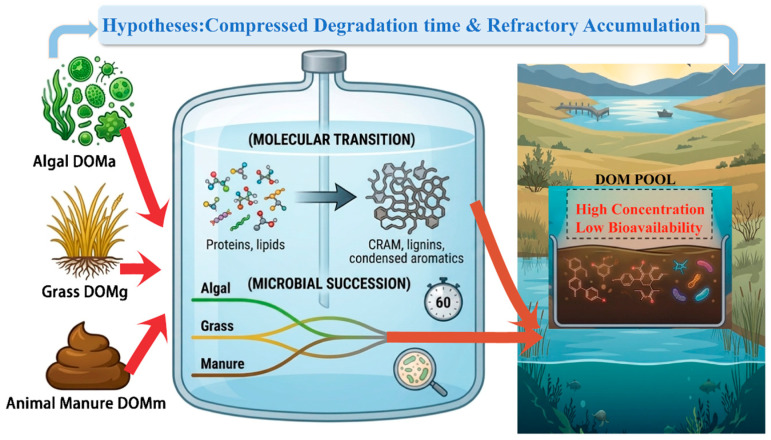
Conceptual diagram of the hypothesised microbial transformation and accumulation of DOM from different sources in cold-region lakes.

To obtain a better understanding of the abnormal lake water environment phenomena in cold regions, characterised by higher DOM concentrations and lower bioavailability, a lab experiment was carried out using Hulun Lake water. This phenomenon was analysed from the perspective of DOM sources (algal, grass and manure sources), chemical composition, biological availability and bacterial community changes. We hypothesised that after different sources of DOM enter the lake, the labile components, such as proteins and lipids, are rapidly utilised and transformed into refractory components through microbial action. These, together with the microbially less available recalcitrant components, such as lignins and condensed aromatics, accumulate within the lake. Subsequently, through long-term biogeochemical processes and influenced by factors such as lake morphology and ice cover, a stable carbon pool is formed, ultimately leading to an increase in organic matter content (Figure 1).

## 2. Materials and Methods

### 2.1. Site Description

**Figure 2:**
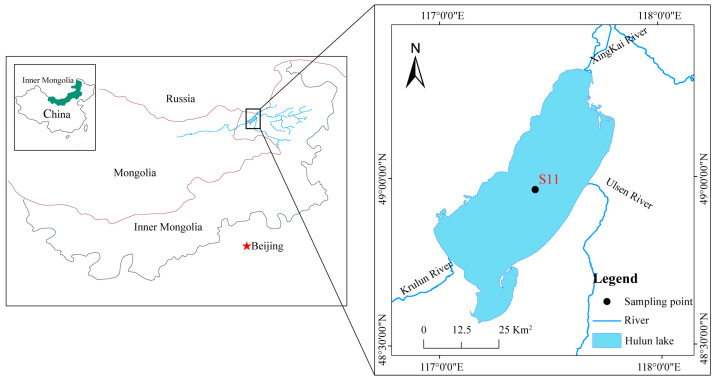
Location of Hulun Lake and the sampling site.

Hulun Lake is located in a cold zone with an annual mean air temperature of −0.24 °C, an ice-covered period of nearly 6 months, and a max ice thickness of approximately 1.3 m [21]. It has an area of 2237.1 km^2^ (2023) and an average depth of 5.7 m (Figure 2). The basins of the lake’s three inflowing rivers are all vast areas of grassland and forest. It is a typical shallow grassland lake with a water quality state that fluctuates between moderate and heavy eutrophication [20].

### 2.2. Test Preparation

First, 100 L of the lake water was collected at the middle layer in the centre part of Hulun Lake in May 2023. The water sample was subsequently preserved in acid-washed polyethylene containers and then transported to the laboratory on the same day. The experimental sample was first filtered using a 0.45 μm pore-size Waterman GF/F membrane (Whatman, Maidstone, UK) to remove larger particles, zooplankton, and phytoplankton and retain bacteria. Then, the filtered sample was maintained in a dark environment for three days, during which the bacteria acclimated to the novel environment [27].

Regarding DOM sources, photosynthetic microorganisms were selected as a typical autochthonous substrate in August 2022 because they are highly susceptible to cyanobacteria blooms in Lake Hulun in the summer [24]. The dominant grass species (*Stipa krylovii Roshev*, *Cleistogenes squarrosa* and *Salsola collina Pall.*) and manure from the main livestock (cattle, horses and sheep) were collected as representative samples of external DOM substrates in the lake basin. Impurities in the samples were removed by screening. The manure and algae samples were treated by lyophilisation, and the grass samples were oven-dried at 65 °C until a constant mass. Detailed extraction of DOM from different sources is described in Text S1.

### 2.3. Experimental Design

The experiments were carried out at the Inland Lakes Research Institute of Northern Cold and Arid Regions, Hulunbeier, Inner Mongolia, China (119°47′19″ E, 49°10′02″ N). To investigate the bioavailability of different sources of DOM in cold-zone lakes, the following treatments were set up in triplicate: different DOM source solutions (DOMa, DOMg, DOMm, and DOMgm) were added to treated lake water samples from Lake Hulun; untreated lake water samples were used as the control group. The DOC concentration in the control group was approximately 17 mg/L. DOMa, DOMg, and DOMm experimental groups were controlled at 40 to 48 mg/L, and DOMgm was controlled at approximately 71 mg/L. Each sample was incubated in a 500 mL glass bottle, and the vessel mouth was covered with a high-temperature-resistant tissue sealing film (BKMAMLAB, Changde, China). All samples were incubated for 60 days at a constant temperature (20 ± 1 °C) in the dark [28]. The samples were shaken sufficiently (every two days) to ensure adequate oxygenation and maintained between 7 and 8 mg/L during the experiment.

The experiment was conducted as a sacrificial experiment, and samples were collected at days 0, 2, 5, 10, 20, 40, and 60. A total of 105 DOM samples were collected throughout the experiment [7 (time point) × 4 (group) × 3 (triplicate)]. DOC concentration, DO, pH, UV–Vis, and 3D-EEMs were measured for each sample. First, 200 mL samples were collected at the beginning (day 0), middle (days 5 and 10), and end (day 60) of the treatment for bacterial community analysis. Additional samples (50 mL) were collected at the beginning (day 0), middle (day 5), and end of treatment (day 60) for Fourier transform ion cyclotron resonance mass spectrometry (FT-ICR MS) analysis.

### 2.4. DOM Characterisation

Samples were filtered through 0.22 μm membrane filters (Jin Teng, PES, Tianjin, China) and kept at 4 °C in the dark before DOC, absorption spectroscopy, EEM fluorescence spectra, and FT-ICR MS measurements.

#### 2.4.1. DOC, CDOM Absorbance, Fluorescence, and Parallel Factor Analysis (PARAFAC)

DOC was analysed using a total organic carbon analyser (TOC-L CPN, Shimadzu Corporation, Kyoto, Japan).

A UV–vis spectrophotometer (PERSEE, Beijing, China) was used to measure the absorption spectra of the samples from 200 to 800 at intervals of 1 nm. Milli-Q ultrapure water was used for zero calibration. The corrected absorption coefficient $a_{254}$ was used to quantify the abundance of chromogenic DOM (CDOM).

(1) $$a_{254}=2.303\times A_{254}/L$$

where $A_{254}$ is the absorbance at 254 nm, and *L* is the optical path (0.01 m).

A fluorescence spectrophotometer (Lab Solutions RF-6000, Shimadzu Corporation, Kyoto, Japan) was used to determine the fluorescence spectra, and the EEM fluorescence spectra were calibrated using Milli-Q water to minimise the effect of instrumental and Raman scattering on the fluorescence spectra. The samples were diluted with Milli-Q ultrapure water until the UV absorbance at 254 nm was less than 0.1 to reduce the fluorescence quenching effect. The scanning wavelength ranges were from 200 nm to 450 nm for the excitation wavelength (Ex), with intervals and excitation bandwidths of 2.0 nm and 5.0 nm, respectively, and from 250 nm to 600 nm for the emission wavelength (Em), with intervals and emission bandwidths of 1.0 nm and 5.0 nm.

EEM fluorescence spectra data were analysed by operating the PARARAC model using the DOMFluor toolbox in MATLAB 2022a software. First, the Rayleigh scattering region (emission wavelength-excitation wavelength <20 nm) was set to zero to eliminate the influence of primary and secondary Rayleigh scattering on the spectra. After removing the outliers by the leverage test, the mathematical model was constructed for 2~7 components sequentially, and the reliability of the model was verified using split-half analysis and Tucker’s congruence coefficient [3].

In this study, five fluorescent components were identified using the EEM-PARAFAC model (Figure S1), and the five fluorescent fractions were compared using the OpenFluor database. The criterion for matching was set at 95% similarity. C1 (Ex/Em: 330/422 nm) is associated with biological activity and the traditional M peak. It is commonly found in seawater as well as in agriculturally influenced catchments [29,30]. C2 (Ex/Em:260, 360/450 nm) is associated with the traditional peaks A and C. It is widespread in surface water environments and is mainly influenced by in situ production and microbial degradation [30,31,32]. C3 (Ex/Em: 320, 390/500 nm) consists of large hydrophobic compounds, with terrestrial and microbial inputs predominating [31,33]. C4 (Ex/Em: 280/400 nm) is a microbially derived, humic-like, relatively aliphatic compound of low molecular weight [34,35]. C5 (Ex/Em: 275/305, 340 nm) is a protein-like component with two excitation peaks representing tyrosine-like and tryptophan-like substances, respectively [36].

#### 2.4.2. FT-ICR MS Measurement

A Fourier transform ion cyclotron resonance mass spectrometer (FT-ICR MS, Bruker SolariX, Bremen, Germany) was used to characterise the molecular composition of DOM in the samples. The ion source was an electrospray ion source (ESI) in negative ion mode. The main detection parameters were continuous injection, an injection rate of 120 μL/h, a capillary inlet voltage of 4.0 kV, an ion accumulation time of 0.06 s, an acquisition mass range of 100~1600 Da, a sampling point number of 4 M 32-bit data, and a time-domain signal superposition of 300 times in order to improve the signal-to-noise ratio. The instrument was calibrated with 10 mmol/L sodium formate prior to sample detection and internal standard calibration with soluble organic matter (known molecular formula) after sample detection. After correction, the mass errors of the assays were all less than 1 ppm. The molecules were classified into four categories based on the presence or absence of heteroatoms N and S in the molecular formula (CHO, CHON, CHONS, and CHOS) and into seven classes based on the H/C and O/C of the molecular formula (lipids (O/C: 0~0.3; H/C: 1.5~2.0), proteins (O/C: 0.3~0.67; H/C: 1.5~2.2), lignins (O/C: 0.3~0.67; H/C: 0.7~1.5), carbohydrates (O/C: 0.67~1.2; H/C: 1.5~2.4), unsaturated hydrocarbons (O/C: 0~0.3; H/C: 0.7~1.5), condensed aromatics (O/C: 0~0.67; H/C: 0.2~0.7) and tannins (O/C: 0.67~1.0; H/C: 0.5~1.5)). Based on the elemental ratios and DBE, CRAMs were identified by 0.3 < DBE/C < 0.68, 0.2 < DBE/H < 0.95 and 0.77 < DBE/O < 1.75 [9,37].

#### 2.4.3. DOM Decomposition

Changes in DOC concentration and CDOM over time were used to estimate DOM fractions (readily biodegradable, slowly biodegradable, and refractory biodegradable) according to Hopkinson et al. [28]. Biodegradation of DOM (final to initial DOC in %) (Equation (2)) follows the pseudo-first-order degradation kinetic equation (Equation (3)). The model can be fitted to different bioavailable fractions of DOM based on the different degradability and degradation rates of DOM. In addition, refractory biodegradability is expressed as *f_c_* (Equation (4)). MATLAB 2022b was used for non-linear fitting, and the R^2^ value after correction was used to reflect the goodness of fit.

(2) $$Degraded\;DOM\;(final\;to\;initial\;DOC\;in\;\%)=({DOC}_{0}-{DOC}_{t})/{DOC}_{0}$$

(3) $$Degraded\;DOM\;(final\;to\;initial\;DOC\;in\;\%)=f_{a}\times \left(1-e^{-k_{1}\times t}\right)+f_{b}(1-e^{-k_{2}\times t})$$

(4) $$f_{c}=1-({DOC}_{0}-{DOC}_{60})/{DOC}_{0}$$

(5) $$t_{1/2}=(ln2)/K$$

where ${DOC}_{0}$ is the initial DOM concentration, and ${DOC}_{t}$ is the concentration that remains after DOM degradation at a given time (t); *f_a_* and *f_b_* are the fractions of readily and slowly biodegradable; $k_{1}$ and $k_{2}$ are the first-order rate biodegradation constants of readily and slowly biodegradable DOM; and t represents the time (days). The half-life ($t_{1/2}$) for different DOM components was calculated using Equation (5).

### 2.5. Bacterial Community

Samples from each experimental group were filtered using 0.22 μm filter membranes, which were stored at −80 °C for measurement. The DNA of the samples was extracted using a DNA extraction kit (MO BIO Laboratories, Carlsbad, CA, USA), and the diluted genomic DNA was used as a template with 338F (ACTCCTACGGGGGAGGCAGCAG) and 806R (GGACTACHVG GGTWTCTAAT) as primers for PCR amplification of the bacterial 16S rRNA gene. This was followed by fluorescence quantification and Illumina high-throughput sequencing. Sequencing results were processed using Qiime (v1.9.0) for quality control, OUT clustering, species annotation and homogenisation.

The similarity coefficient was used to quantify the similarity between DOM samples from different sources and the control group during the experiment. The formula is as follows:

*Jsi* = *c*/(*a* + *b*) (6)

where *Jsi* is the Jaccard similarity coefficient, *c* is the formula for the same formula in both samples (A and B), *a* is the number of formulas in sample A, and *b* is the number of formulas in sample B.

### 2.6. Statistical Analysis

One-way ANOVA and other basic plots were carried out using SPSS Statistics 27 and Origin 2024, respectively. Other statistical analyses were performed in R version 4.4.1, including principal coordinate analysis of bacterial communities and canonical correlation analysis (CCA) of bacterial communities with DOM molecules.

## 3. Results

### 3.1. Biodegradation of DOM

**Figure 3:**
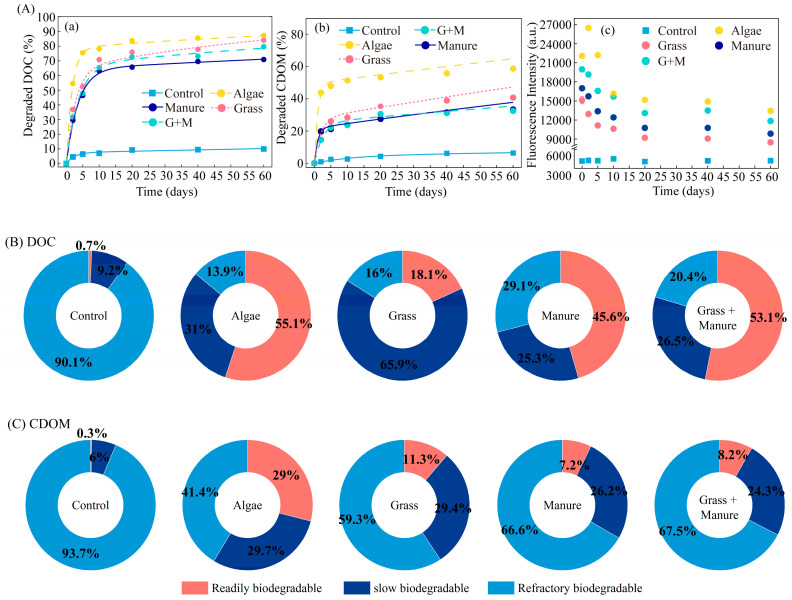
DOM removal rates (**A**) and percentages of bioavailable components (**B**,**C**) during the biodegradation of each potential DOM source. (**A**) (**a**,**b**) Changes in the removal rates of total DOM and CDOM and the fitting results of pseudo-first-order degradation kinetic equations; however, the change in fluorescence intensity (**c**) did not follow the pseudo-first-order degradation kinetic equation, so no fitting curve was generated. (**B**,**C**) The proportion of bioavailable DOM components.

The amount of bioavailable DOM in the water of Lake Hulun was only 9.94% (as DOC). DOMa had higher bioavailability than DOMg and DOMm, and DOMg was slightly higher than DOMm (Figure 3A). During 60 days of incubation, the degraded DOC was 86.11%, 85.45% and 70.87% for DOMa, DOMg and DOMm, respectively. In the first 5 days, the degraded DOC of DOMa, DOMg and DOMm was 75.5%, 54.45% and 46.40%, respectively, with DOMa being significantly higher than DOMg and DOMm (one-way ANOVA, *p* > 0.001). Between 5 and 60 days, the degradation of DOC was 10.61%, 31.63% and 24.47% for DOMa, DOMg and DOMm, respectively. The characteristics of the changes in CDOM, FDOM and DOC were similar, and the degradation of CDOM and FDOM was significantly less than that of DOC. The degradation of CDOM (32.48% to 58.64%) was only approximately 50% of DOC, and the degradation of FDOM (27.91% to 42.39%) was even less. Notably, the fluorescence intensity of DOMa increased significantly at 2d.

Biodegradable DOM and CDOM from different sources of DOM were fitted with the pseudo-first-order degradation kinetic equation model based on their different degradation rates (Figure 3B; Table S1). The bioavailable fraction of DOM in Lake Hulun was found to be approximately 10% of the total DOC. The bioavailable fraction of CDOM was only 6.3%. DOC fitting results showed that DOMa had the highest percentage of readily biodegradable components at 53.8%, while DOMm and DOMg had 45.6% and 18.5%, respectively. However, DOMg contained 67.6% of slowly biodegradable fractions. The results of CDOM fitting for DOMa were distinct from those of DOMg and DOMm. DOMa had the highest percentage of readily biodegradable fractions at 29%, which was approximately three times higher than DOMg (11.3%) and DOMm (7.2%). The proportion of slowly biodegradable fractions was similar, at 29.7% (DOMa), 29.4% (DOMg) and 26.2% (DOMm). The half-lives of the readily biodegradable fraction of DOM ranged from 1.18 to 2.53 days for all experimental groups, with half-lives of 49.51, 77.02 and 198.04 days for the slowly biodegradable fractions of DOMa, DOMg and DOMm, respectively (Table S1). Interestingly, DOMgm had a significantly higher readily biodegradable fraction of DOC (53.1%) than DOMg and DOMm alone, whereas CDOM showed no significant difference. These colourless organic matters have often been neglected in past investigations of the optical features of DOM.

### 3.2. Changes in Molecular Composition

**Figure 4:**
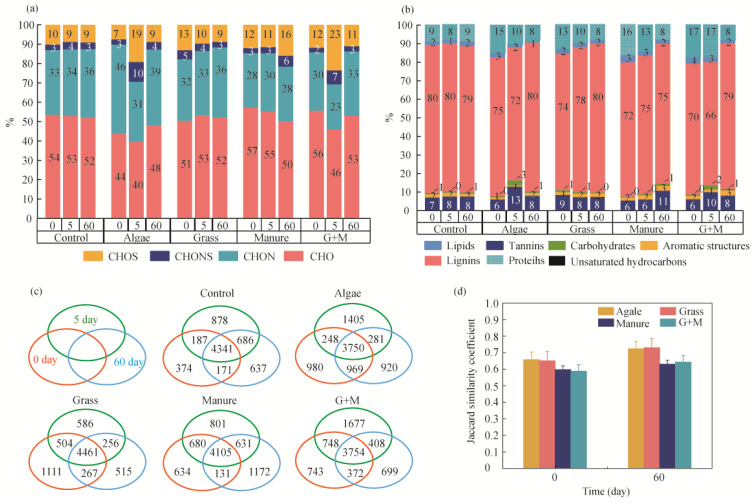
Changes in molecular composition and ratios during biodegradation of DOM. (**a**) Changes in elemental composition; (**b**) changes in compound composition; (**c**) changes in the number of shared molecules; (**d**) changes in Jaccard similarity.

In the water body of Lake Hulun, dissolved organic matter (DOM) was predominantly composed of lignin, accounting for approximately 80% of the total DOM. Proteins and tannins followed, contributing 9% and 7%, respectively. The relative abundance of elemental composition followed the order CHO% > CHON% > CHOS% > CHONS% (Figure 4a). Four sources of DOM—denoted as DOMa, DOMm, DOMg, and DOMgm—were found to primarily enhance the protein fraction. Specifically, the molecular count percentage of proteins increased from a common baseline of 9% to 15% (DOMa), 13% (DOMm), 16% (DOMg), and 17% (DOMgm) (Figure 4b). Concurrently, the total number of molecular formulas rose from an initial baseline of 432 to 840 (DOMa), 787 (DOMg), 885 (DOMm), and 928 (DOMgm). In terms of intensity-weighted percentage, the protein-related signals increased from a common baseline of 5.18% to 12.37% (DOMa), 8.27% (DOMg), 13.38% (DOMm), and 14.38% (DOMgm) (Table S2). Overall, the molecular composition across all four DOM sources was dominated by CHO- and CHON-containing compounds. Notably, DOMg exhibited a relatively high proportion of CHOS- and CHONS-containing formulas, accounting for 17.63% of its total molecular formulas.

The common molecular formula of DOM among the experimental groups consisted of approximately 80% lignin compounds, followed by tannins (6.3% to 7.8%) and protein compounds (approximately 8.3% to 9.9%) (Figure 4c). The DOM-specific molecular formulas were also dominated by lignin. DOMm, DOMg, DOMgm and DOMa were dominated by lignin, with the control containing 68% of lignin. There was a dramatic increase in the number of lignin molecules in biodegradable DOM and a decrease in the percentage of proteins. The lignin molecular formulas of DOMa, DOMg, DOMm, and DOMgm increased by 23.06%, 22.31%, 8.37%, and 15.46%, respectively. For DOMm, excluding lignin, the increase in tannins was highest, at 22.58%. According to the Jaccard similarity coefficient, the molecular composition of various DOM sources exhibited increased similarity to the control (Figure 4c). The *Jsi* coefficient of DOMg increased from the initial value of 0.65 to 0.73, and the coefficient of DOMa increased from 0.66 to 0.72 (Figure 4d). The similarity of the molecular composition of DOMm showed a smaller change, from an initial value of 0.60 to 0.63.

**Figure 5:**
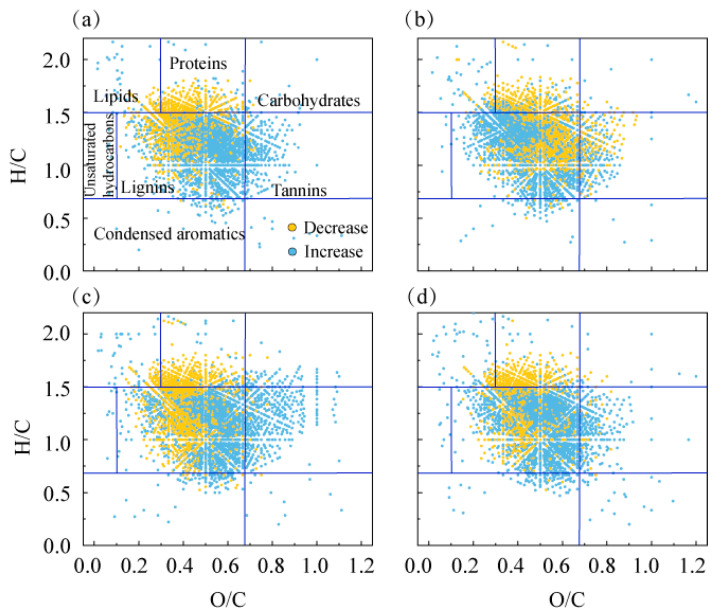
Changes in DOMa (**a**), DOMg (**b**), DOMm (**c**) and DOMgm (**d**) molecules during the 60-day microbial incubation.

In order to evaluate the contribution of microbial processes to the molecular transformations of different DOM sources, the changes in the relative intensities of molecular components of each DOM source were analysed (Table S2). The compounds consumed by the microbiological processes of each DOM source were mainly proteins and lipids, and the compounds produced were mainly lignin and tannins (Figure 5). The relative molecular intensities of DOMg, DOMa, DOMm, and DOMgm proteins decreased by 3.32, 6.22, 7.00, and 7.93%, respectively. The relative intensities of lignin molecules increased by 4.82%, 5.39%, 2.85%, and 7.18%, respectively. In addition, the number of molecular formulas of CRAMs representing newly formed molecules significantly increased during degradation. The relative molecular intensities of CRAMs in DOMa and DOMg increased by 12.64% and 5.26%, respectively.

### 3.3. Changes in Bacterial Community

**Figure 6:**
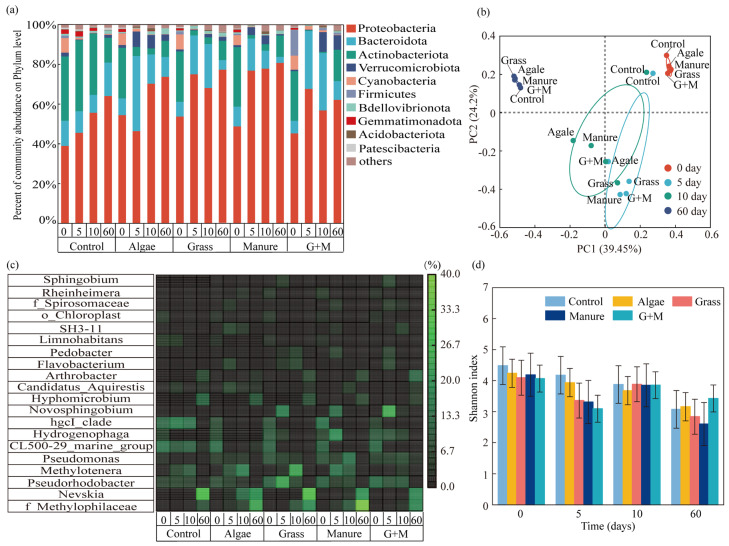
Bacterial community analysis. Relative abundance of microbial communities at the phylum level (**a**). Bacterial principal coordinates analysis (**b**). Relative abundance of bacterial communities at the genus level (**c**). Plot of changes in microbial diversity index (**d**).

The sample data set consisted of 45,288 sequences and 4989 OTUs. At the phylum level, Proteobacteria (39.15%), Actinobacteria (32.39), Bacteroidetes (12.66%) and Cyanobacteria (7.32%) were the most abundant in the initial control sample (Figure 6a). The addition of different sources of DOM solution increased the percentage of Proteobacteria and decreased Actinobacteria and Bacteroidetes. The relative abundance of Proteobacteria in DOMa, DOMg, DOMm and DOMgm increased to 54.64%, 53.96%, 48.98% and 45.47%, respectively, while Actinobacteria decreased to 25.52%, 25.28%, 29.92% and 24.9% and Bacteroidetes decreased to 8.38%, 7.42%, 10% and 6.36%, respectively. The relative abundance of Proteobacteria continued to increase with incubation time, and DOMa, DOMg and DOMm were 73.9%, 77.37% and 80.9% at 60 days. The relative abundance of Bacteroidetes showed a trend of increase and then decrease with time. DOMa, DOMg and DOMm reached the highest relative abundance at 5 days, at 37.83%, 19.56%, and 16.19%, respectively. DOMg had the highest abundance of 22.19% at 10 days. The trend of the Actinobacteria phylum was opposite to that of Bacteroidetes. Overall, the dominant phyla at 60 days were Proteobacteria (62.4~80.9%), Bacteroidetes (3.1~10.01%), Actinobacteria (8.16~15.78%), and Verrucomicrobia (1.89~7.23%). These dominant bacterial phyla are well-known organic-matter-degrading microorganisms [9].

Bacterial communities were further analysed at the genus level (Figure 6c). The highest numbers in the initial control group were *CL500-29_marine_group* (14.67%), *hgcI_clade* (12.59%), *Pseudorhodobacter* (6.64%), *Hydrogenophaga* (6.18%), *Limnohabitans* (5.03%), *Norank__f__norank__o___Chloroplast* (3.84%), *Pseudomonas* (3.25%) and *Candidatus_Aquirestis* (1.18%). At the end of the experiment, the dominant genera in the experimental group were identified as *unclassified_f_Methylophilaceae* (18.17%~37.99%) and *Nevskia* (18.13%~32.22%), followed by *Hyphomicrobium* (4.67%~10.66%), *Arthrobacter* (2.49%~12.66%), and *Candidatus_Aquirestis* (2.04%~4.04%). Principal coordinate analysis was used to compare bacterial community characteristics during DOM degradation (Figure 6b). In general, the bacterial communities exhibited greater dispersion across experimental groups on days 5 and 10, whereas they demonstrated increased aggregation on days 0 and 60.

### 3.4. Biodegradation of DOM Affected by Bacterial Community

**Figure 7:**
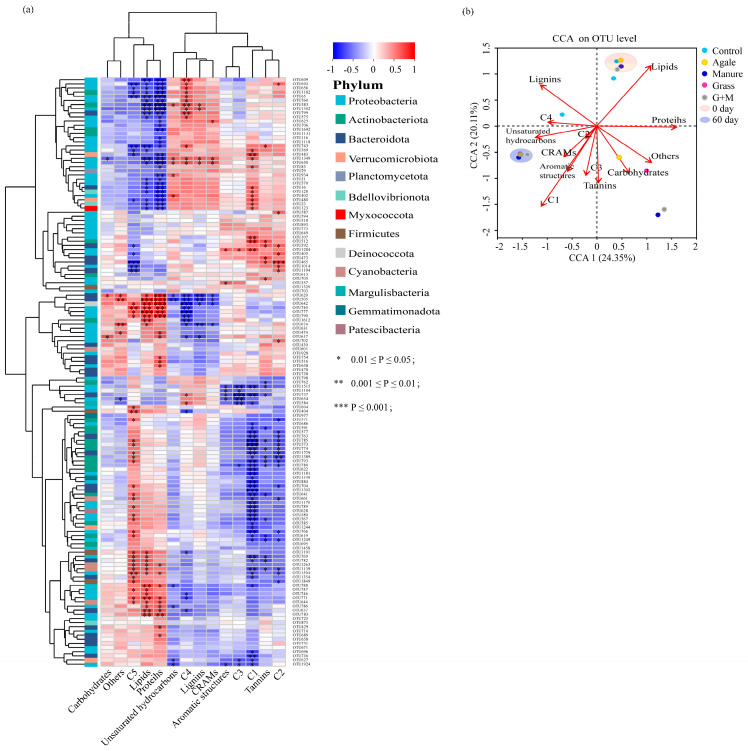
Analysis of the relationship between bacterial taxa and DOM components. (**a**) Spearman correlation heat map between DOM and bacteria. (**b**) CCA of DOM fractions and microbial communities from different sources (OTU level).

DOM components (fluorescence intensity and relative intensity of molecular composition) were correlated with the dominant bacterial species (OTU abundance > 0.001) and analysed by clustering (Figure 7a). The DOM fractions were clustered into three categories: the first being unstable DOM fractions dominated by C5, proteins and lipids; the second being refractory DOM fractions dominated by C4, lignin and CRAMs; and the third being DOM fractions dominated by C3, C1, C2, and tannins. The correlations between the first type of DOM fractions and the second and third types of DOM fractions with OTUs showed significant differences. Proteins, lipids and C5 showed significant correlations (*p* < 0.05) with a wide range of microorganisms, with Proteobacteria (60%) and Bacteroidota (18%) dominating. Transformation of the C1 fraction involved the largest number of microbial species, with Proteobacteria (38%), Actinobacteriota (23.6%), and Bacteroidota (16.4%) dominating. Proteobacteria (OTU1349, OTU674, OTU650, OTU617, and OTU1502), Bacteroidota (OTU505, OTU620), Deinococcota (OTU642), Actinobacteriota (OTU583) and other bacterial communities were significantly associated with a variety of other DOM components. In addition, in order to explore the transformation process of DOM utilisation by bacteria, random forest was used to screen bacterial genera with significant effects on each fluorescence component and molecular formula. Analyses showed that the process of microbial utilisation of lignin involved more complex microbial species than lipids and proteins, with *Hyphomicrobium* and *Flavobacterium*, *Candidatus_Aquirestis*, *Pseudomonas*, *o__Chloroplast*, *f__Spirosomaceae* and other genera being extensively involved in the transformation of lignin compounds (Figure S2).

CCA was performed on the operational taxonomic unit (OTU) level for components and bacterial communities at different time points across different DOM sources (Figure 7b). CCA1 and CCA2 explained 24.35% and 20.11% of the molecular composition of bacteria and DOM samples, respectively. On day 0, positive correlations with lipids and proteins and negative correlations with compounds such as CRAMs, condensed aromatics, tannins, and C1 were observed for the microbial communities of each experimental group. At day 60, the correlations between bacteria and DOM fractions showed the opposite characteristics to those at day 0. Lignin, protein, and carbohydrates were significantly correlated with the bacterial community at day 5. In contrast, bacterial communities were more aggregated on days 0 and 60 across the experimental groups, and the relationship between bacteria and DOM components was similar among different sources. At day 5, the bacterial communities were more dispersed, and the bacterial communities of DOMg, DOMm, and DOMgm were affected by multiple DOM components compared to DOMa.

## 4. Discussion

### 4.1. Biodegradation Processes of Different DOM Sources

Our study demonstrated the proportions of bioavailable components from different DOM sources in cold-region lakes and estimated their reaction times. The total bioavailable DOM from different sources differed, but the temporal pattern of change was similar for all DOM sources during incubation (Figure 3A). The readily degradable fractions of the various DOM sources were rapidly utilised over the course of one to three days. During this period, changes in the DOC concentration primarily coincided with alterations in the relative abundance of protein and lipid molecules [13,38]. As the readily degradable components were exhausted, the slowly biodegradable components were gradually utilised. The half-life of slowly biodegradable fractions of organic matter from DOMm and DOMg was significantly longer than that of DOMa. After 60 days of microbiological treatment, a significant portion of DOC was still observed (13.9% to 29.1%), which is often difficult to utilise by microorganisms and accumulates in the aqueous environment.

In addition to the sources of DOM, the composition of the microbial community plays an important role in the transformation of DOM [39]. Our study further found that the final products of DOM from different sources under microbial action tend to be similar to the control group (Figure 4d). This increase in similarity is mainly due to the depletion of unstable DOM (H/C > 1.5, compounds such as proteins, lipids, etc.) and the increase in nascent refractory DOM by microbial action [40]. CRAM is a major contributor to DOM refractory components in the aquatic environment [41,42]. All DOM sources contributed significant fractions of CRAMs to the aqueous DOM, and both the molecular formulas and relative peak intensities of CRAMs in the newly generated molecules increased with incubation time. This also supports the idea that microbial metabolism contributes to the formation of refractory DOM in the aquatic system [43,44]. The yields of CRAMs generated by the different DOM sources were very different. Among the newly generated molecules, DOMa generated the highest number of CRAMs, DOMg generated the highest percentage of CRAMs, and DOMm generated an increasing number of CRAMs with a decreasing percentage. The readily biodegradable DOM fraction is an important driver of diversity in DOM transformation, as readily biodegradable and slowly biodegradable DOM fractions favour the growth of a more diverse microbiota [13,44]. The DOMa, DOMm and DOMgm experimental groups were all observed to have more nascent DOM molecular formulations, especially at day 5 when the number of newly generated molecules was significantly higher (Table S3). The proportion of lignin and CRAMs was substantially increased in the newly generated molecules, explaining why persistent DOM in lakes tends to exhibit similar molecular characteristics.

### 4.2. Response of Bacterial Community to Different DOM Sources

Bacterial community assembly tends to change in response to changes in the quantity and quality of DOM in the environment [45], and different sources of DOM lead to distinct processes of bacterial community succession (Figure 7a, b). At the initial stage of degradation (0 days), a large number of readily biodegradable components of the DOM source can be used by a wide range of bacteria, reducing competition between bacteria and allowing more stochastic community succession [13,46]. As the readily biodegradable compounds are depleted, the percentage of slow biodegradable compounds (5 and 10 days) increases, and resource and energy constraints result in increased deterministic bacterial selection [14]. Differences in the utilisation of slowly biodegradable components among different DOM sources may lead to differences in bacterial communities (Figure 6d and Figure 7b). DOMa has a higher proportion of readily biodegradable components and higher sensitivity to bacterial action than DOMg and DOMm [14,40]. Thus, the Shannon index after 5 days was significantly higher than that of the other experimental groups. As the readily biodegradable components are depleted, the selective pressure is further increased, forcing the matching of effective bacteria and available resources [13]. The bacterial community structure evolves towards similar stable assemblages, as the residual DOM components and properties are similar across DOM sources.

The environmental effects of bacterial communities are not realised by the superposition of their individual parts but by the synergy of their multiple taxa [45]. Specifically, the bacterial utilisation of macromolecular carbon sources requires additional community members to facilitate degradation for other bacteria [13,39,43]. The process of lignin utilisation by bacteria involves more bacterial genera than proteins and lipids (Figure S2), among which *Hyphomicrobium*, *Flavobacterium*, *Spirosomaceae* and other genera are all characterised by lignin conversion [47,48]. However, the abundance of these genera was relatively low among the dominant genera in the initial control group (Figure 6). This may indicate that the bacterial utilisation of DOM in natural lakes is very different from our experimental results. Bacterial degradation of DOM in aquatic ecosystems is a continuous reaction process [15], but such a continuous reaction may be difficult to achieve in natural lakes, especially for most eutrophic lakes. Sufficient available resources in the high trophic state give microorganisms a wider choice of carbon sources, and the initiation time for slowly biodegradable components is difficult to determine. Furthermore, interannual environmental variation in cold lakes can strongly influence the succession of microbial community structure [49], which in turn affects the dynamic association between DOM and microorganisms.

### 4.3. Causes of High DOM and Low Bioavailability

Our study verified the view of previous researchers that inputs from terrestrial sources are the cause of organic matter accumulation in lakes [21,22,50]. Terrestrial sources of DOM (DOMg and DOMm) have more slowly biodegradable components that are difficult for microorganisms to use in a short period of time and that accumulate in the lake. Terrestrial inputs are the main source of DOM in most cold-region lakes. Eutrophication of lakes and seasonal algal blooms exacerbate organic matter accumulation [7,44,51]. DOM from high trophic-state lakes tends to be dominated by high molecular weight, unsaturated, condensed or polycyclic aromatic compounds, which are difficult to utilise by microorganisms [7,51]. In addition, eutrophication causes seasonal outbreaks of algal blooms, and the process of microbial transformation of algal-derived organic matter produces a large number of refractory DOM [9,40,44]. In recent years, algal blooms have occurred frequently in lakes in the region [24,25]. Hulun Lake frequently experiences cyanobacterial blooms [52]. Specifically, such blooms have intensified since the 2010s. In 2022, the bloom area reached 1970.55 km^2^, covering approximately 88.07% of the lake surface [24,52]. Cyanobacteria, particularly Dolichospermum circinale, are the dominant bloom-forming taxa, leading to extraordinary accumulation of refractory DOM by microorganisms. In addition, microbial utilisation of DOM may be inhibited by the environmental characteristics of alternating freezing and thawing [53,54]. The process of lake freezing and thawing contributes to changes in physicochemical environmental factors such as water temperature, hydrodynamics, and DO [55,56,57], which affect the structure and activity of phytoplankton and bacterial communities [58], exacerbating the accumulation of organic matter by reducing the time during the year that organic matter can be efficiently utilised. In recent years, due to the reduction in regional water resources [59], longer water exchange cycles have led to annual accumulation [60] and the complex “processing” of the lake DOM.

In summary, the formation of high organic matter concentration and low bioavailability in cold-zone lakes is the result of a combination of factors. However, with the further shortening of the ice cover period of cold lakes due to global warming [61,62], the stabilisation mechanism of organic matter in cold lakes will be greatly altered, thus accelerating the process of global carbon cycling.

## 5. Conclusions

In this study, laboratory incubation experiments were conducted to investigate the microbial degradation processes and accumulation mechanisms of DOM from different sources in grassland lakes in cold and arid regions. To the best of our knowledge, this is the first systematic study on the microbial degradation kinetics and structural characterisation of DOM derived from livestock manure in grassland ecosystems. DOM bioavailability is highly dependent on its source. The initial composition of DOM from different sources leads to differences in transformation pathways. However, microbial depletion of labile DOM components and the concurrent generation of refractory components promote the formation of similar end products. Our results suggest that the high organic matter content and low bioavailability observed in cold-region lakes may be primarily attributed to their unique organic matter sources, the region’s alternating freeze–thaw climatic characteristics, and long-term accumulation. For northern grassland lakes, measures such as strengthening watershed discharge management, restoring grassland vegetation, reducing soil erosion, strictly enforcing the grass-livestock balance, and enhancing water exchange should be implemented to effectively reduce organic pollution. This study did not consider the effects of light, seasonal variations, or dynamic inputs, and the widely present soil-derived DOM was not included. Therefore, the findings have certain limitations.

## Data Availability

The original contributions presented in this study are included in Supplementary Materials. Further inquiries can be directed to the corresponding author.

## Supplementary Materials

The following supporting information can be downloaded at https://www.mdpi.com/article/10.3390/toxics14040347/s1, Text S1: Extraction process of the DOM from different sources; Figure S1: 3D-EEM fluorescence maps of different DOM substrates; Figure S2: Effect of microbial dominant species on lignins (a), lipids (b), proteins (c), C1 (d), C4 (e), and C5 (f); Table S1: The pseudo-first-order rate constant (*k*, day^−1^), half-life (day), and correlation coefficient (*R*^2^) of the potential DOM source; Table S2: Variation in the number of molecular formulas and the corresponding relative peak intensities of DOM formulas from different sources over 60 days; Table S3: Variation in the number of newly generated formulas and the corresponding relative peak intensities of DOM formulas from different sources over 60 days.
