# Supplementary material for: Understanding the Causes of High Organic Matter with Low Bioavailability in Cold-Zone Lake Water: A Case in Hulun Lake
Source: Toxics. 2026 Apr 20;14(4):347. doi: 10.3390/toxics14040347 (PMC13120219; doi:10.3390/toxics14040347)
Supplement: Supplementary file 1 [file toxics-14-00347-s001.zip › toxics-4234789-supplementary.pdf]

Supporting Information:

Understanding the Causes of High Organic Matter with Low
Bioavailability in Cold-Zone Lake Water: A Case in Hulun Lake

Yulong Tao

Hulunbuir Academy of Inland Lakes in Northern Cold & Arid Areas, Hulunbuir 021000, China; taoyulong_1982@163.com

Text S1. Extraction process of the DOM from different sources

The different DOM source solutions were obtained as follows. **Algae:** Here, 20 g of freeze-dried algae powder was mixed with 1 L of Mill-Q water. Then, the solution was ultrasonicated for 60 min, followed by shaking at 220 r/min for 12 h and static settling (20 °C, 12 h). Finally, the algal extract was obtained by centrifugation of the algal water supernatant (4000 r/min, 45 min). The obtained algal solutions were also passed through a 0.22 μm filter membrane (Waterman, GF/F) to remove particulate matter before performing the experiments. Grass solutions and manure solutions were prepared by the same process as the algal extract. **Grass:** Here, 80 g of a mixed grass powder sample (Stipa krylovii Roshev, Cleistogenes squarrosa, and Salsola collina Pall. mixed at a weight ratio of 2:1:1) was added to 1 L of ultrapure water. **Manure:** Here, 100 g of a mixed manure powder sample (cattle manure, horse manure, and sheep manure mixed at a weight ratio of 2:1:1) was added to 1 L of ultrapure water.

Supplementary Figures

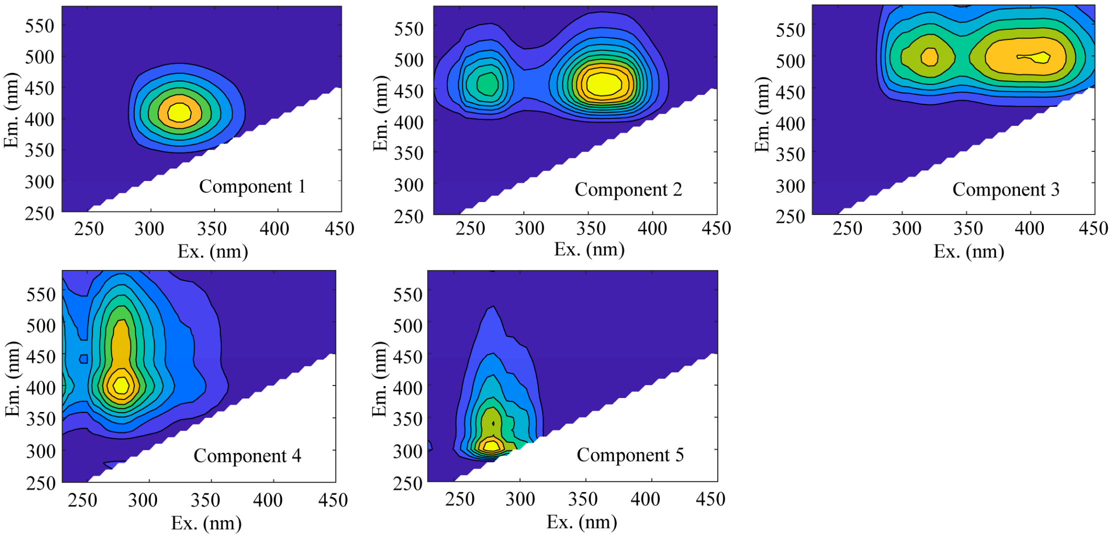

Figure S1. 3D EEM fluorescence maps of different DOM substrates.

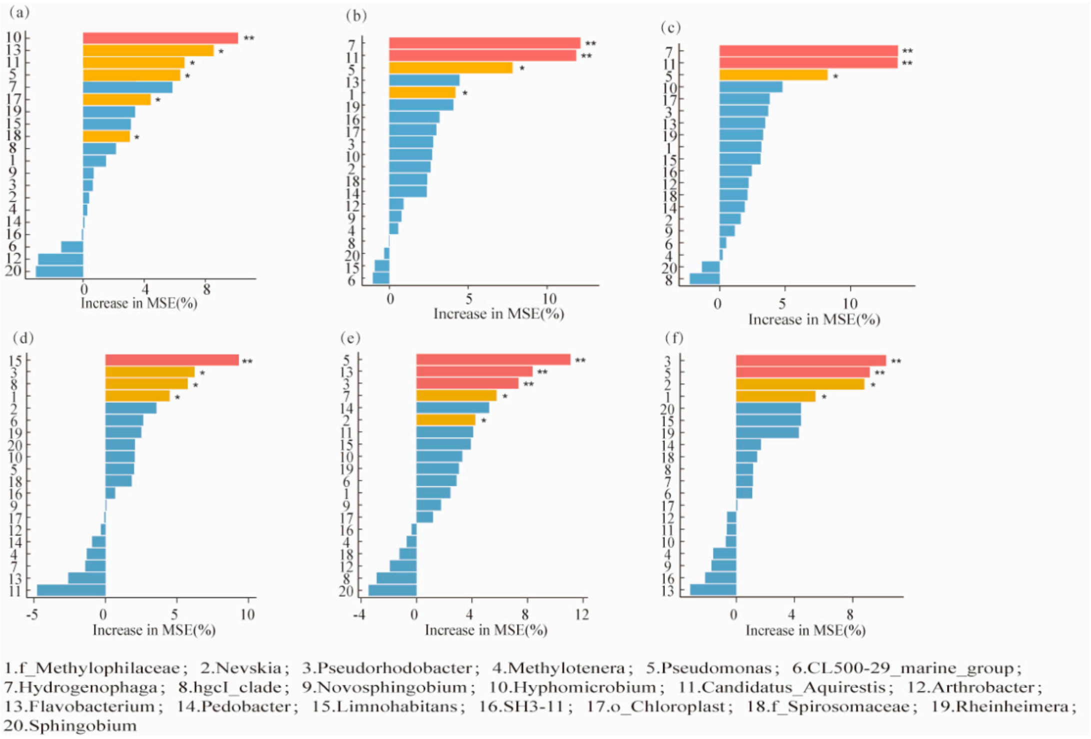

Figure S2. Effect of microbial dominant species on lignins (a), lipids (b), proteins (c), C1 (d), C4 (e), and C5 (f).

Supplementary Tables

Table S1. The pseudo-first-order rate constant (k, day^{-1}), half-life (day), and correlation coefficient (R^2) of the potential DOM source.

DOM Solution	Rate<br>% of total DOC	readily (f_a)<br>(% of total Rate)	slow (f_b)	K_1<br>(day^{-1})	K_2<br>(day^{-1})	Half-life 1<br>(day)	Half-life 2<br>(day)	R^2
DOC degradation fitting parameters								
Control	9.94	7.45	92.55	0.405	0.0005	1.71	1386.29	0.97
DOMa	86.11	63.97	36.03	0.360	0.014	1.93	49.51	0.98
DOMg	84.08	21.49	78.1	0.585	0.009	1.18	77.02	0.99
DOMm	70.87	64.3	35.7	0.278	0.0035	2.49	198.04	0.98
DOMgm	79.60	66.68	33.32	0.274	0.007	2.53	99.02	0.97
CDOM degradation fitting parameters								
Control	6.33	4.75	95.25	0.087	0.0003	7.97	2310.49	0.96
DOMa	58.64	49.47	50.53	0.99	0.0028	0.70	247.55	0.99
DOMg	40.70	27.70	72.3	0.525	0.0037	1.32	187.34	0.98
DOMm	33.36	21.75	78.25	0.99	0.0029	0.70	239.02	0.98
DOMgm	32.48	25.26	74.74	0.387	0.0018	1.79	385.08	0.98

Table S2. Variation in the number of molecular formulas and the corresponding relative peak intensities of DOM formulas from different sources over 60 days.

Type	Day	Class	All formulas	tannins	Aromatic structures	Unsaturated hydrocarbons	Carbohydrates	lignins	lipids	proteins	CRAMs
Control	0	Number	4928	360	89	4	27	3940	76	432	2913
		Intensity-weighted percentage	100%	4.60%	0.23%	0.01%	0.09%	89.44%	0.46%	5.18%	58.63%
	5	Number	5936	465	133	9	23	4747	78	481	3418
		Intensity-weighted percentage	100%	5.45%	0.29%	0.01%	0.09%	89.17%	0.37%	4.62%	56.67%
	60	Number	5665	444	88	5	34	4471	112	511	3282
		Intensity-weighted percentage	100%	5.26%	0.19%	0.01%	0.11%	88.67%	0.48%	5.27%	57.48%
DOMa	0	Number	5757	365	70	6	28	4301	147	840	3548
		Intensity-weighted percentage	100%	4.73%	0.19%	0.01%	0.20%	80.97%	1.53%	12.37%	56.68%
	5	Number	5502	728	37	2	146	3947	99	543	1822
		Intensity-weighted percentage	100%	16.14%	0.41%	0.00%	2.10%	70.70%	0.62%	10.03%	42.93%
	60	Number	5762	476	100	10	30	4616	76	454	3306
		Intensity-weighted percentage	100%	6.43%	0.28%	0.01%	0.24%	86.36%	0.53%	6.15%	58.20%
DOMg	0	Number	6154	537	90	3	64	4525	148	787	3329
		Intensity-weighted percentage	100%	6.06%	0.22%	0.01%	0.59%	84.23%	0.63%	8.27%	54.02%
	5	Number	5648	424	121	7	29	4416	101	550	3280
		Intensity-weighted percentage	100%	5.31%	0.34%	0.02%	0.10%	87.95%	0.53%	5.74%	57.34%
	60	Number	5350	411	103	5	19	4293	89	430	3214
		Intensity-weighted percentage	100%	5.19%	0.29%	0.01%	0.07%	89.05%	0.45%	4.95%	59.27%
DOMm	0	Number	5373	305	89	1	21	3893	179	885	3039
		Intensity-weighted percentage	100%	4.43%	0.33%	0.00%	0.13%	80.77%	0.96%	13.38%	55.70%
	5	Number	6033	380	139	3	26	4516	158	811	3319
		Intensity-weighted percentage	100%	4.73%	0.41%	0.00%	0.10%	83.42%	0.62%	10.72%	55.84%
	60	Number	5870	644	154	8	68	4423	93	480	3072
		Intensity-weighted percentage	100%	8.32%	0.47%	0.01%	0.74%	83.62%	0.45%	6.38%	54.56%
DOMgm	0	Number	5447	349	105	6	25	3840	194	928	3007

	Intensity-weighted percentage	100%	5.14%	0.34%	0.01%	0.22%	79.02%	0.89%	14.38%	53.48%
5	Number	6379	654	93	3	137	4242	183	1067	2890
	Intensity-weighted percentage	100%	10.55%	0.27%	0.01%	1.77%	72.72%	0.57%	14.11%	46.48%
60	Number	5074	423	145	9	29	3989	85	394	3024
	Intensity-weighted percentage	100%	6.15%	0.56%	0.03%	0.11%	86.20%	0.51%	6.45%	57.63%

Table S3. Variation in the number of newly generated formulas and the corresponding relative peak intensities of DOM formulas from different sources over 60 days.

Type	Day	All formulas	tannins	Aromatic structures	Unsaturated hydrocarbons	Carbohydrates	lignins	lipids	proteihs	others	CRAMs
Control	0	374	20	18	3	2	236	27	40	28	157
	5	878	68	52	6	9	645	19	62	17	403
	60	637	46	18	2	11	398	48	83	31	294
DOMa	0	980	22	15	3	11	426	86	374	43	356
	5	1405	420	17	3	130	586	47	153	49	330
	60	920	85	45	7	8	699	25	32	19	517
DOMg	0	1111	138	15	3	47	500	80	279	49	317
	5	586	32	43	6	13	377	39	55	21	278
	60	515	33	54	4	4	365	31	35	16	280
DOMm	0	634	19	17	3	10	306	79	200	0	258
	5	801	31	36	1	9	528	45	115	36	408
	60	1172	289	49	6	52	640	35	59	42	430
DOMgm	0	743	30	30	5	12	345	76	212	33	293
	5	1677	329	28	3	116	723	55	359	64	490
	60	699	53	126	8	9	465	36	29	36	354
